# Community supported agriculture plus nutrition education improves skills, self-efficacy, and eating behaviors among low-income caregivers but not their children: a randomized controlled trial

**DOI:** 10.1186/s12966-021-01168-x

**Published:** 2021-08-31

**Authors:** Rebecca A. Seguin-Fowler, Karla L. Hanson, Stephanie B. Jilcott Pitts, Jane Kolodinsky, Marilyn Sitaker, Alice S. Ammerman, Grace A. Marshall, Emily H. Belarmino, Jennifer A. Garner, Weiwei Wang

**Affiliations:** 1 Texas A&M AgriLife Research, 600 John Kimbrough Boulevard, Suite 512, College Station, TX 77843 USA; 2 Department of Population Medicine and Diagnostic Sciences, Master of Public Health Program, Cornell University, S2064 Schurman Hall, Ithaca, NY 14853 USA; 3 Department of Public Health, East Carolina University, 600 Moye Blvd, Lakeside Annex Modular 7, Greenville, NC 27834 USA; 4 Department of Community Development and Applied Economics, University of Vermont, 202 Morrill Hall, Burlington, VT 05405 USA; 5 Ecological Agriculture and Food Systems, The Evergreen State College, 2700 Evergreen Parkway NW, Olympia, WA 98505 USA; 6 Department of Nutrition, Gillings School of Global Public Health, Center for Health Promotion and Disease Prevention, University of North Carolina at Chapel Hill, 1700 Martin Luther King Boulevard, CB#7426, Chapel Hill, NC 27599 USA; 7 Master of Public Health Program, Cornell University, S2074 Schurman Hall, Ithaca, NY 14853 USA; 8 Department of Nutrition and Food Sciences, University of Vermont, 225 B Marsh Life Science, Burlington, VT 05405 USA; 9 School of Health and Rehabilitation Sciences, College of Medicine, John Glenn College of Public Affairs, The Ohio State University, 210N Page Hall, Columbus, OH 43210 USA; 10 Center for Rural Studies, University of Vermont, 206 Morrill Hall, 146 University Place, Burlington, VT 05405 USA

**Keywords:** Community supported agriculture, Cost-offset, Subsidized, Low income populations, Skin carotenoids, Food security, Attitudes, Fruit and vegetable eating behaviors

## Abstract

**Background:**

Adults and children in the U.S. consume inadequate quantities of fruit and vegetables (FV), in part, due to poor access among households with lower socioeconomic status. One approach to improving access to FV is community supported agriculture (CSA) in which households purchase a ‘share’ of local farm produce throughout the growing season. This study examined the effects of cost-offset (half-price) CSA plus tailored nutrition education for low-income households with children.

**Methods:**

The Farm Fresh Foods for Healthy Kids (F3HK) randomized controlled trial in New York, North Carolina, Vermont, and Washington (2016–2018) assigned caregiver-child dyads (*n* = 305) into cost-offset CSA plus education intervention or control (delayed intervention) groups. Following one growing season of CSA participation, changes in children’s diet quality, body mass index (BMI), and physical activity; caregivers’ nutrition knowledge, attitudes, behaviors, and diet quality; and household food access and security were examined using multiple linear or logistic regression, with adjustment for baseline value within an intent-to-treat (ITT) framework in which missing data were multiply imputed.

**Results:**

No significant net effects on children’s dietary intake, BMI, or physical activity were observed. Statistically significant net improvements were observed after one growing season for caregivers’ cooking attitudes, skills, and self-efficacy; FV intake and skin carotenoid levels; and household food security. Changes in attitudes and self-efficacy remained one-year after baseline, but improvements in caregiver diet and household food security did not. The number of weeks that participants picked up a CSA share (but not number of education sessions attended) was associated with improvements in caregiver FV intake and household food security.

**Conclusions:**

Cost-offset CSA plus tailored nutrition education for low-income households improved important caregiver and household outcomes within just one season of participation; most notably, both self-reported and objectively measured caregiver FV intake and household food security improved. Households that picked up more shares also reported larger improvements. However, these changes were not maintained after the CSA season ended. These results suggest that cost-offset CSA is a viable approach to improving adult, but not child, FV intake and household food security for low-income families, but the seasonality of most CSAs may limit their potential to improve year-round dietary behavior and food security.

**Trial registration:**

ClinicalTrials.gov. NCT02770196. Registered 5 April 2016. Retrospectively registered.

**Supplementary Information:**

The online version contains supplementary material available at 10.1186/s12966-021-01168-x.

## Background

Despite the benefits of adequate fruit and vegetable (FV) intake [1], most individuals in the U.S. do not eat recommended amounts [2], with lower intake among individuals with lower socioeconomic status [3, 4]. Findings suggesting that greater FV access is related to higher intake underpin ongoing public health efforts to increase FV intake [5, 6]. One strategy to reduce disparities in FV access and intake is to leverage direct-to-consumer marketing of fresh produce – such as community supported agriculture (CSA) – to reach more low-income households [7, 8].

In the traditional CSA model, members pay for a ‘share’ of a farm’s produce upfront, and then have consistent access to fresh produce throughout the growing season. Most studies have shown the purchase of full-priced CSA to be positively associated with FV intake: CSA members consumed more FV [9, 10], had greater increases in FV intake than non-members [11, 12], and ate more fruits and/or vegetables during the CSA season than before [13–16]. However, some studies did not observe a positive effect [17, 18] or had mixed results [19–22]. Prior research has also found positive associations between CSA purchase and diet quality [14, 15, 17, 19, 23] and healthy eating behaviors [10, 11, 13, 15, 21, 24, 25]. For example, compared to non-members, CSA members reported eating more salads [10], home-cooked meals [11], and family meals [21]; fewer processed snacks [10]; and eating in restaurants less often [21]. CSA participation also may have implications for body mass index (BMI); one study showed a beneficial association between CSA and BMI [23] and another showed none [21].

Among the studies that examined associations between purchase of full-priced CSA and FV intake, diet quality, and healthy eating behaviors, some had small samples (e.g. 50 or fewer participants) [14, 18, 20], did not use a comparison group [13–20, 24, 25], and/or included only data from during or after the CSA season (i.e. no baseline or pre-test) [9, 13, 15, 16, 23–25]. Further, none randomized participants to CSA or examined objectively-assessed FV intake and, as such, intervention-related bias may have influenced results. Two studies of CSA purchasers focused on low-income families [15, 22], but most included primarily middle- and upper-income participants.

A persistent critique of the CSA model is that payment is generally required in advance of the growing season, which may limit participation by low-income families [26, 27]. CSA that provides a *cost-offset* to low-income households (CO-CSA) removes this financial barrier by subsidizing a portion of the share price, eliminating up-front payments, and offering flexible payment plans. Descriptive studies have suggested that CO-CSA participation may positively impact FV access [28, 29] and intake [30, 31]. A pilot study reported that all CO-CSA participants increased the variety of vegetables consumed, learned new ways to prepare vegetables, and liked a new vegetable after CSA participation, but there were no changes in other outcomes [29]. Longitudinal studies of cost-offset (or free) CSA programs more often reported increases in fruit and/or vegetable intake [32–36] than reported no changes [29, 37]. A recent randomized trial comparing CO-CSA with unconditional cash transfer reported improvements in overall diet quality; diet quality subscores for total vegetables, total fruit, whole fruit, and empty calories; and food security status [36].

Similar to studies of full-priced CSAs, many studies of CO-CSA had small samples [27, 29, 31, 37], no comparison group [27, 29, 31, 34, 37], included only data from during/after the CSA season [30, 31, 33], and/or recruited samples not limited to low-income participants [33, 34]. Only two studies used a randomized study design [36, 37] and none examined effect based upon objectively-assessed FV intake. Three of the CO-CSA programs provided FV at no cost to the participants [27, 32, 37], and one provided home-delivered shares [32], both of which may limit their generalizability. Only two studies incorporated nutrition education for participants beyond general CSA tip sheets or newsletters [35, 37].

This study aims to fill gaps in the understanding of the effects of CO-CSAs on food security, dietary intake, and related outcomes. The overall aim of the Farm Fresh Foods for Healthy Kids (F3HK) randomized controlled trial was to assess the effect of CO-CSA participation coupled with tailored nutrition education for low-income families with children living in rural and micropolitan communities [38]. The primary aim of this trial report is to assess the short-term net effect of CO-CSA plus nutrition education on children’s diet quality, particularly FV intake. Secondary aims are to examine net effects of CO-CSA plus nutrition education on children’s BMI, physical activity, and sedentary behavior, as well as on caregivers’ food and nutrition skills, knowledge, attitudes, beliefs, and diet quality, as well as household food security. Tertiary aims are to test whether any observed short-term effects were durable after the CSA season ended (i.e. lasted into the next spring), and to estimate the role of intervention dose in understanding observed intervention effects.

## Methods

### Intervention

Development of the F3HK intervention was informed by formative interviews conducted with caregivers and children from low-income households in target communities [39, 40], CSA members and farmers [41], and cooperative extension nutrition educators [42], as well as analysis of observational data [30, 31]. The focus of the F3HK intervention was a summer CSA membership of 15–24 weeks length (mean = 21 weeks) plus nutrition education [43]. Nine farms offered multiple CSA share sizes from which caregivers could select the option that best suited their needs and preferences (mean = 7.5 items/week) [43]. Shares were offered at half-price and caregivers paid weekly, on average $13, with money or Supplemental Nutrition Assistance Program (SNAP) benefits throughout the season [43]; research funds paid the other half of the price to the farm before the CSA summer season began. SNAP benefits are provided by the U.S. federal government to eligible low-income individuals and families (household income at or below 130% of the poverty line and a maximum of $2250 in financial assets) [44]. The average monthly benefit per person in 2018 was $124.50 [45]. If paying with their monthly SNAP benefits, families were choosing to use their SNAP funds to pay for the CSA share instead of any type of food from other authorized venues (e.g. grocery stores). Fidelity of the CSA was high with respect to produce quality and pick-up site functionality as observed and recorded by local research coordinators [43]. Participation level was high; on average, caregivers picked up their CSA share 88% of weeks enrolled [43].

Families in the CO-CSA plus nutrition education group were offered kitchen tools and education classes. Caregivers selected 2–4 larger kitchen tools from among the following: food processor, crockpot, stockpot, large cutting board, chef’s knife, salad spinner, and reusable grocery bag. Selected items were shipped to their home at the beginning of the CSA season [38]. Adults and children also were invited, but not required, to attend nine in-person CSA-tailored education classes offered locally [38]. Social Cognitive Theory [46], the Dietary Guidelines for Americans [47], the Physical Activity Guidelines for Americans [48], and agricultural calendars in each state [49–54] guided the design of the CO-CSA tailored curriculum [38]. Classes featured seasonal produce via food tasting, demonstrations, hands-on cooking activities, handouts, and recipes; two of the lessons involved field-based learning via grocery store and farm tours; and three lessons taught the use of a vegetable peeler, vegetable scrub brush, or paring knife which participants were allowed to keep [38]. Adherence to the curriculum was high as reported by educators and as observed and recorded by state research coordinators [43]. Most caregivers (77%) and children (54%) attended at least one class, but almost no one attended all classes [43].

### Study design

The F3HK study (protocol published elsewhere [38]) used a randomized controlled trial with one-way cross-over of control to intervention after 1 year. Participants enrolled in wave one (2016) were assigned to a two-year CO-CSA plus nutrition education intervention and in wave two (2017) were assigned to a one-year CO-CSA plus nutrition education intervention. One-to-one random assignment occurred following baseline assessments and was generated by Qualtrics in blocks of four within each of the 12 farm communities in New York, North Carolina, Vermont, and Washington. Multiple staff members reported assignments to participants, thereby reducing the likelihood of assignment prediction by study staff. Once assigned, neither participants nor staff were blinded to assignment for logistical reasons. Our study was approved by the University of Vermont (protocol ID: CHRBSS 16–393) and Cornell University (protocol ID: 1501005266) Institutional Review Boards.

### Sample

**Fig. 1:**
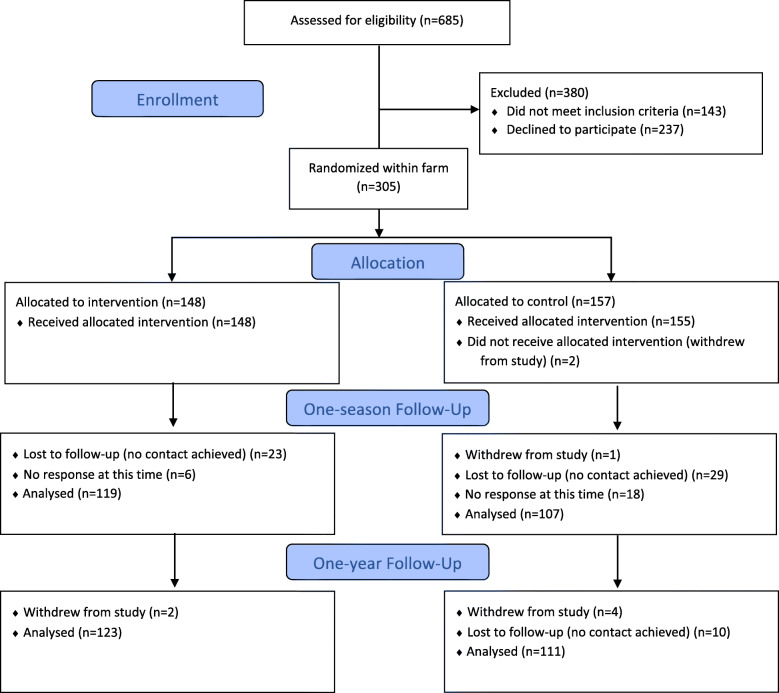
Consolidated Standards of Reporting Trials Statement diagram showing study flow for Farm Fresh Foods for Healthy Kids (F3HK)

Flyers, newspapers, and social media were used to advertise the study opportunity, and study staff directly recruited at schools, churches, libraries, community service organizations, and at local events from January through June 2016 and 2017. Participants were also identified via “word of mouth.” Caregivers completed a brief electronic screening tool on a tablet or were later screened over the telephone. Households were eligible if they resided in a participating community, included a child aged 2–12 years old who was willing to participate, met guidelines for low income (< 185% federal poverty level), and had not participated in CSA for at least 3 years [38]. This last criteria was included because we wanted to recruit participants who were ‘naïve’ to CSA programs. A total of 685 caregivers were screened for eligibility and 542 (79.1%) were determined to be eligible; see Fig. 1.

Once determined eligible, caregivers had to agree to spend their own money or SNAP benefits on weekly CSA payments and complete the baseline survey, and an age-eligible child had to be willing to participate as well. Of 542 eligible caregiver-child dyads, 305 (56.3%) enrolled. The first dyad was enrolled on March 4, 2016 and the last on June 20, 2017. The target sample size of 300 was designed to detect a difference of one-third serving of vegetables between groups (power = 0.80, *p* = 0.05, two-sided test), a targeted effect size slightly smaller than those reported in a systematic review of the effect of nutrition education on FV intake among low-income adults [55].

### Data collection

Data collection included online surveys that were completed by caregivers with input from children for children’s diet measures, as well as physical measurements of skin carotenoids and children’s height and body weight, which were obtained by research staff. Caregivers also completed three 24-h recalls for, or with, their child. Because 24-h recalls can be time consuming, we expected fewer recall responses, presented this activity as optional, and provided separate compensation specifically tied to this activity completion. Data analyzed here were collected at baseline (spring), one-season follow-up (fall; mean = + 5.02 months, SD = 0.77) and survey data only at one-year follow-up (spring; mean = + 9.69 months, SD = 0.81). Caregiver compensation included: $25 for each online survey completed, $50 each time three 24-h recalls were completed, and $10 for skin carotenoid measures. Caregivers who completed surveys at all three time points also earned a $25 bonus. Children were compensated $10 for skin scanning and $10 for height and weight measurements. Most participants (76.7%) were retained through one-year follow-up measures.

### Primary outcomes

Primary outcomes of children’s dietary intake were measured using validated tools: online survey measures of FV intake and frequency of intake of sweets, salty snacks, and sugar sweetened beverages (SSBs); physical measurement of skin carotenoids; and online completion of 24-h dietary recall data for children. Survey items were completed by the caregiver with 70.0% of children age six or older assisting. FV intake with and without juice (cups/day) was measured using the National Cancer Institute’s (NCI) All-Day Fruit and Vegetable Screener (FVS) [56]. Sweets, salty snacks, and SSB consumption were measured using the Beverage and Snack Questionnaire, version 2 (BSQ2) [57, 58]. The BSQ2 was adapted to remove questions regarding FV and 100% juice intake to avoid overlap with the FVS, and to omit distinctions in response options for consumption that occurred in-school and out-of-school. Monthly intake frequency of five items of candy, sweet baked goods, and ice cream were summed into “sweets”; three salty snacks items into “salty snacks”; and six items including regular sodas, sweetened coffee/tea and drinks, and flavored milks into “SSBs.”

Caregivers completed two or three online dietary recalls for, or with, the child through the NCI Automated Self-Administered 24-h recall system [59]. At baseline, 10.6% of those who completed dietary recalls completed only two recalls and at one-season follow up 3.2% only completed two recalls. Recalls provided additional measures of the child’s FV intake with and without juice (cups/day), as well as solid fat (g), sodium (mg), and added sugar (tsp) intake. From 24-h recall data, the child’s overall diet quality was assessed by the total Healthy Eating Index-2015 (HEI) [60, 61], and mean total energy intake as a percentage of child’s estimated energy requirements (EER) given sex, age, and reported days of physical activity (described below) [47].

Skin carotenoids were assessed in-person with the Pharmanex© Biophotonic Scanner S3 (NuSkin Enterprises, Provo, UT, USA) via resonance Raman spectroscopy (RRS) by trained research staff. Assessment of skin carotenoid score using RRS is a valid method to approximate FV intake in children [62–64] and is sensitive to increases in FV intake among youth [65]. The mean of two or three RRS measurements were used as an objective biomarker of children’s FV intake.

### Secondary outcomes

Secondary outcomes included child’s body weight and activity levels; caregiver’s skills, knowledge, attitudes, behaviors, and dietary intake; and household food security. Trained research staff measured the height and weight [66] of each child from which we calculated BMI percentile [67]. Each child’s physical activity was reported by caregivers as days of physical activity (≥60 min) in the past week [68]. Sedentary activity was reported as hours per school day the child spent watching TV and hours per school day spent playing video games [68].

Caregivers’ ability to select, store, and prepare CSA produce was measured using the 14-item Cooking Techniques and Meal Preparation Self-efficacy Scale (range 1 to 5) [69]. Seven additional questions about specific skills included in the F3HK curriculum were added to create an expanded 21-item scale [38]. Caregivers’ ability to substitute FV for energy-dense foods was assessed using questions about monthly frequency of preparing nine different FV snacks for children, which were highlighted in the F3HK curriculum [38]. Responses were summed to create measures of the monthly frequency of preparing fruit, vegetable, and total FV snacks. Data also were collected to measure caregivers’ ability to prepare foods to minimize added solid fat and sugar but were not analyzed due to low response.

Caregivers’ knowledge, attitudes, and beliefs (KAB) about FV were collected via the online survey. Knowledge of FV recommendations was assessed with ordinal response options subsequently combined into indicators of > 5 cups FV or less, and FV covering half or more of a dinner plate or less. The four-item Negative Cooking Attitude Scale [69] was used to measure caregivers’ attitudes towards cooking (range 1 to 5); higher scores indicate greater dislike of cooking. The 11-item General Nutrition Knowledge Belief Score collected beliefs about the importance of a healthy diet and other eating behaviors (range 1 to 4) [70]. Self-efficacy was collected via the 4-item Self-Efficacy for Eating/Cooking Fruits and Vegetables Scale (range 1 to 5) [69].

Caregiver’s diet was assessed via the survey using the NCI-FVS and BSQ2 as described above. Skin carotenoids were measured for caregivers using RRS technology, which has been validated in adults [71–74] and responds to a high-carotenoid diet [75]. Home FV availability and accessibility were assessed using multi-item scales (range 1 to 4) [76]. Affordability and physical accessibility of FV were each assessed using one Likert-type question (range 1 to 5). Household food security was measured using the 6-item Short Form of the US Department of Agriculture Food Security Survey Module [77]. Households were classified as food secure if they experienced none or one of the indicators of insufficiency in the past 30 days.

### Participation level

Participation level was measured as the number of weeks that participants picked-up the CSA share based on logs kept by farm staff, and as the number of CSA-tailored classes attended by the caregiver based on attendance logs kept by the educators. At both follow-up time points, caregivers reported whether or not they participated in a CSA outside of the intervention (e.g. a winter CSA), which was explored as a potential source of bias.

### Analyses

Normality of continuous outcomes was checked by examining histograms and checking skewness and kurtosis statistics. Single time-point measures with skewness > 1.0 were transformed to approximate normality (e.g. Ln FV intake; cube-root sweets, salty snacks, and SSBs; Ln added sugar; square-root solid fat; cube-root sodium; Ln sedentary hours; Ln FV snack preparation frequency). Baseline (spring) data were available for almost all participants for almost all variables: only 1.6% of data were missing for any of the survey outcomes. Skin carotenoid status was missing for 13.8% of participants at baseline, largely due to unforeseeable delays in the shipping of equipment. As expected, fewer participants (80.7%) provided at least two 24-h recalls at baseline.

Follow-up (fall) data were available for the majority of participants. Fifty-five participants (18.0%) withdrew or were lost to follow-up and another 24 (7.9%) did not provide data at that time but were responsive later in the study. The primary concern was that data may not be missing at random (MAR) and, in the context of a health behavior intervention trial, that participants with worse baseline health behaviors might be most likely to drop-out or not report. To explore this potential bias, we compared baseline values for all outcomes for respondents and non-respondents at one-season (fall) follow-up. For eight of 38 outcomes (21.1%), cases missing follow-up data were significantly different from those not missing data, but there was no consistent direction to the associations -- missing follow-up data was associated with two *healthier* behaviors and six *less healthy* behaviors at baseline, and unrelated for the other 30 outcomes. These findings do *not* provide strong evidence that systematic bias resulted from missing data.

Multiple imputation was used because it is the most accurate approach to estimating missing data and standard errors that account for both variability due to sampling and to imputation itself [78]. Imputations followed standardized, rigorous procedures [79], included auxiliary variables [80], used 30 imputations [78], and employed both standard and hierarchical approaches. Baseline and one-season follow-up data were imputed using a fully conditional specification (FCS) standard approach, which is considered an unbiased and robust approach to imputation of missing dichotomous and continuous data [81]. Logistic and linear regression were used for categorical and continuous variables, respectively [PROC MI, SAS software (SAS Institute Inc., Cary, NC, USA; version 9.4)]. Due to computational limitations, data were multiply imputed in four groups: 1) survey measures of diet and physical activity for children and caregivers; 2) survey measures of caregivers’ KAB and behaviors, and household food access, availability, and security; 3) objective measures with survey measures of diet and physical activity as additional auxiliary variables; and 4) outcome measures derived from 24-h recalls, also with survey measures of diet and physical activity as auxiliary variables. Participants who *never* provided any 24-h recalls (*n* = 59, 19.3%) were excluded from imputation and analysis of these outcomes. All models also included key characteristics as auxiliary variables [78]: random assignment group, state; caregiver race (white or not), age, education (college degree or not), and health status; and age, sex, and health of the child. Data for the three time points were similarly imputed using a FCS linear mixed-effects latent normal approach, which performs well for the estimation of regression model parameters in longitudinal data [78] and Blimp version 2 [82].

Analysis of change scores from baseline was the primary method of net effect estimation for continuous outcomes given high correlation between baseline and follow-up outcomes [83]. Changes in continuous outcomes were confirmed to approximate normality based on a skewness statistic under 2.0, or visual examination of residuals against the predicted means to confirm symmetry and even distribution. Change scores could not be calculated for binary outcomes and were modeled as the observed value at the time-point of interest. Regression estimates were considered significant at *p* < 0.05.

#### One-season effects

Estimates of changes from baseline (spring) to one-season follow-up (fall) for intervention group relative to control were examined for all complete cases (n varies) using multiple linear or logistic regression (for continuous and binomial variables, respectively), with adjustment for baseline value. These net one-season effects also were estimated using an intent-to-treat (ITT) framework, multiply imputed data (*n* = 305 survey and objective measures, and *n* = 264 24-h recalls), and PROC MIANALYZE in SAS.

#### Durability and participation level

Net effects from baseline (spring) through one-year follow-up (spring) were examined similarly using multiply imputed longitudinal data and multi-level linear or logistic regression. An interaction between intervention and season (spring vs. fall) was included in models to test for differences in the intervention net effect at one-season compared to one-year follow-up. Number of weeks of CSA pick-up and number of lessons attended were used to predict short-term change in outcomes within the intervention group.

#### Sensitivity analyses

The ITT analysis to assess one-year net intervention effect was conducted two more times to control for potential bias due to enrollment in a CSA outside the intervention: once assuming that the 77 missing responses got an outside CSA and once assuming they did not.

## Results

Table 1 presents baseline characteristics of caregivers, children, and households. Overall, there were no notable differences in baseline characteristics across random assignment groups.

**Table 1:** Baseline characteristics of enrollees in the F3HK intervention trial in four U.S. states, 2016-2017

		Intervention			Control		
		n	Count (Mean)	% (SD)	n	Count (Mean)	% (SD)
**CAREGIVER CHARACTERISTICS**							
Age		148	(35.7)	(7.5)	157	(36.5)	(8.4)
Sex	Male	148	5	3.4	157	3	1.9
	Female		143	96.6		154	98.1
General health	Excellent – very good	148	51	34.5	157	54	34.4
	Good		69	46.6		57	36.3
	Fair – poor		28	18.9		46	29.3
Marital status	Married	148	63	42.6	157	69	43.9
	Separated, divorced, or widowed		42	28.4		34	21.7
	Never married		31	20.9		37	23.6
	A member of an unmarried couple		12	8.1		17	10.8
Highest year of school	High school or less	148	24	16.2	157	36	22.9
	Technical or vocational school		7	4.7		7	4.5
	Some college		41	27.7		41	26.1
	College graduate		56	37.8		61	38.9
	Graduate or professional degree		20	13.5		12	7.6
Employment status	Employed	148	69	46.6	157	71	45.2
	Out of work		15	10.1		22	14.0
	A homemaker		55	37.2		53	33.8
	Student/retired		9	6.1		11	7.0
Race	American Indian/ Alaskan Native	148	1	0.7	157	3	1.9
	Asian/Pacific Islander		2	1.4		2	1.3
	Black		23	15.5		19	12.1
	White		112	75.7		120	76.4
	Multiracial		7	4.7		9	5.7
	Not one of the above		3	2.0		4	2.5
Hispanic	Yes	148	9	6.1	157	10	6.4
	No		139	93.9		147	93.6
**CHILD CHARACTERISTICS**							
Age		148	(6.1)	(3.0)	157	(6.2)	(3.0)
Gender	Male	148	65	43.9	157	81	51.6
	Female		83	56.1		76	48.4
General health	Excellent – very good	148	119	80.4	157	124	79.0
	Good		23	15.5		25	15.9
	Fair – poor		6	4.1		8	5.1
**HOUSEHOLD CHARACTERISTICS**							
State	New York	148	45	30.4	157	48	30.6
	North Carolina		37	25.0		40	25.5
	Vermont		34	23.0		37	23.6
	Washington		32	21.6		32	20.4
# Adults in household	1 adult	148	48	32.4	157	54	34.4
	2 adults		87	58.8		85	54.1
	3 or more adults		13	8.8		18	11.5
# Children in household	1 child	148	43	29.1	157	45	28.7
	2 children		53	35.8		61	38.9
	3 children		30	20.3		34	21.7
	4 or more children		22	14.9		17	10.8
Annual household income	Less than $9,999	147	22	15.0	155	34	21.9
	$10,000 - $14,999		19	12.9		11	7.1
	$15,000 - $19,999		16	10.9		14	9.0
	$20,000 - $24,999		18	12.2		25	16.1
	$25,000 - $34,999		34	23.1		28	18.1
	$35,000 - $49,999		31	21.1		30	19.4
	$49,999 - $74,999		7	4.8		13	8.4
Household receive WIC in the past month?	No	148	87	58.8	157	100	63.7
	Yes		61	41.2		57	36.3
Household receive SNAP in the past month?	No	147	77	52.4	155	74	47.7
	Yes		70	47.6		81	52.3

Complete case analysis revealed improvement in children’s FV intake without juice as measured by 24-h recalls (+ 0.35 cups/day, *p* < 0.05) (Supplemental Table 1, Additional File 1). In the ITT analyses with multiply imputed data, no significant net effects on children’s dietary intake remained (Table 2). Likewise, no net effects of F3HK participation were observed on the secondary outcomes of children’s BMI, physical activity, or sedentary time in ITT analyses.

**Table 2:** One-season effects of the F3HK intervention relative to control, 2016-2017

	Intervention			Control			Net Effect After Adjustment for Baseline	Sig./CI
		Baseline	One-season Change		Baseline	One-season Change		
	*n*	Mean/%	Mean/%	*n*	Mean/%	Mean/%		
**PRIMARY OUTCOMES**								
**Child’s FV Intake** (cups/day)								
Total, NCI-FVS	148	4.28	-0.11	157	3.75	-0.31	+0.56	0.20
Total without Juice, NCI-FVS	148	3.19	0.04	157	2.85	-0.34	+0.58	0.09
Total, ASA24	135	2.80	0.27	129	2.82	-0.05	+0.35	0.10
Total without Juice, ASA24	135	2.43	0.28	129	2.44	-0.10	+0.37	0.06
**Child’s Mean Skin Carotenoid RRS Score**	148	35605.70	-880.67	157	34744.34	-1646.72	+985.58	0.48
**Child’s Intake of SSBs and Processed Snacks**								
Sweets (times/month), BSQ2	148	34.09	-2.40	157	46.79	-14.11	+7.50	0.19
Salty Snacks (times/month), BSQ2	148	23.69	1.60	157	29.31	-0.01	-0.37	0.93
SSBs (times/month), BSQ2	148	39.64	-4.51	157	50.57	-6.62	-0.07	0.99
Solid Fat Intake (g), ASA24	135	31.24	1.73	129	30.35	2.70	-0.61	0.78
Sodium Intake (mg), ASA24	135	2596.12	202.52	129	2668.54	60.09	+102.24	0.40
Added Sugar Intake (tsp), ASA24	135	11.56	-0.41	129	10.88	0.65	-1.05	0.27
**Child’s Overall Diet Quality**								
Total HEI Score, ASA24	135	60.93	-0.59	129	60.63	-1.47	+1.03	0.49
Energy as %EER^a^, ASA24	135	111.90	1.50	129	114.14	-0.07	+0.50	0.91
**SECONDARY OUTCOMES**								
**Child’s BMI-for-age** (percentile)	148	64.72	-0.53	157	69.83	0.16	-1.03	0.58
**Child’s Physical Activity** (days/week)	148	5.46	0.26	157	5.13	0.36	+0.09	0.69
**Child’s Sedentary Behavior** (hours/day)								
Time Watching TV on a School Day	148	1.31	-0.23	157	1.44	-0.10	-0.17	0.20
Time Playing Video Games on School Day	148	0.76	-0.13	157	0.82	-0.16	+0.01	0.89
**Caregiver’s Ability to Select, Store, and Prepare CSA Produce**								
Cooking Techniques and Meal Preparation Self-Efficacy, original 14-item (scale 1-5)	148	3.94	0.19	157	3.76	0.06	+0.20	**<0.01**
Cooking Techniques and Meal Preparation Self-Efficacy, expanded 21-item (scale 1-5)	148	3.75	0.29	157	3.56	0.06	+0.29	**<0.01**
**Caregiver’s Ability to Substitute FV for Energy-Dense Foods** (times/month)								
Preparing FV as Snacks for Children	148	69.33	18.34	157	65.34	0.59	+19.22	**<0.01**
Preparing Fruit as Snacks for Children	148	38.64	5.62	157	34.05	0.14	+7.92	**<0.01**
Preparing Vegetables as Snacks for Children	148	30.69	12.72	157	31.29	0.45	+12.09	**0.04**
**Caregiver’s Knowledge, Attitudes, and Beliefs About FV**								
Knew Adult FV Recommendation Was 5+ Cups/Day (%, OR)	148	47.3	2.2	157	30.1	7.3	1.16	0.89, 1.51
Knew FV Recommendation Was ≥ Half of Dinner Plate (%, OR)	148	74.9	11.2	157	70.0	8.1	1.29	0.90, 1.86
General Nutrition Knowledge Belief (scale 1-4)	148	3.23	-0.01	157	3.15	0.09	-0.08	0.11
**Negative** Cooking Attitudes (scale 1-5)	148	2.09	-0.21	157	2.19	0.12	-0.35	**<0.01**
Self-Efficacy for Eating and Cooking Fruits and Vegetables (scale 1-5)	148	3.75	0.33	157	3.62	0.01	+0.38	**<0.01**
**Caregiver’s FV Intake** (cups/day)								
Total, NCI-FVS	148	4.38	0.34	157	4.03	-0.35	+1.10	**0.02**
Total without Juice, NCI-FVS	148	3.66	0.43	157	3.38	-0.39	+1.01	**0.03**
**Caregiver’s Mean Skin Carotenoid RRS Score**	148	29518.36	2441.53	157	29038.82	-776.84	+3312.16	**0.01**
**Caregiver’s Intake of SSBs and Processed Snacks** (times/month)								
Sweets, BSQ2	148	33.85	-8.31	157	37.63	-2.78	-6.78	0.30
Salty Snacks, BSQ2	148	15.44	-1.10	157	24.10	-2.63	-2.31	0.52
SSBs, BSQ2	148	39.25	-2.30	157	51.11	3.66	-9.61	0.22
**Availability and Accessibility of FV in the Home**								
Availability of FV in the Home (scale 1-4)	148	3.46	0.13	157	3.37	0.07	+0.11	**0.02**
Accessibility of FV in the Home (scale 1-4)	148	3.35	0.15	157	3.22	0.11	+0.11	0.20
How Easy to Financially Afford FV (Likert-type 1-5)	148	2.80	0.25	157	2.70	0.18	+0.12	0.27
How Easy to Access FV (Likert-type 1-5)	148	3.91	0.35	157	3.77	0.20	+0.23	**0.03**
**Household is Food Secure, FFSM** (%, OR)	148	42.6	12.0	157	44.0	-5.1	1.67	**1.18, 2.37**

Significance of intervention status on change in outcome from baseline was tested using multiple linear regression for continuous variables and multiple logistic regression for dichotomous variables. All p-values presented are from models adjusted for the baseline value of the dependent variable

^a^Calculated using baseline age and assuming a moderate level of physical activity

There were, however, significant one-season changes observed among secondary outcomes related to caregivers and households in both complete case and ITT analyses. All five measures of caregivers’ skills improved significantly by the end of the summer CSA season. Both of the scales measuring cooking techniques and meal preparation self-efficacy improved in the intervention group relative to control (+ 0.20, *p* < 0.01 [14-item scale] and + 0.29, *p* < 0.001 [21-item scale]). There were also statistically significant positive net effects on caregiver behaviors such as preparing FV as snacks for children (+ 19.22, *p* < 0.01), as well as preparation of fruit (+ 7.92, *p* < 0.01) and vegetables alone (+ 12.09, *p* < 0.05). F3HK showed no net effect on caregivers’ knowledge of FV recommendations and general nutrition, but negative cooking attitudes (− 0.35, *p* < 0.001) and self-efficacy for eating and cooking FV (+ 0.38, *p* < 0.001) improved. Caregivers’ FV intake also improved in the F3HK intervention group relative to control, assessed by self-reported FV intake with and without juice (+ 1.10, *p* < 0.05 and + 1.01, *p* < 0.05), and supported by net effects on objective measures of skin carotenoids (+ 3312.16, *p* < 0.05). There were no observed effects on caregivers’ consumption of sweets, salty snacks, or SSBs.

We also observed a positive one-season net effect of F3HK on two of the four measures of FV availability and access: the availability of FV in the home (+ 0.11, *p* < 0.05) and ease of access to FV (+ 0.23, *p* < 0.05). Additionally, household food security (OR 1.67, *p* < 0.01) showed net improvement.

Some one-season net effects on secondary outcomes were maintained into the following spring (when no CSA FV were being received; Table 3). Net one-season intervention effects were maintained at one-year follow-up for scales of cooking techniques and meal preparation self-efficacy (+ 0.28 < *p* < 0.01 and + 0.34, *p* < 0.01), as well as negative cooking attitudes (− 0.25, *p* < 0.01) and self-efficacy for eating and cooking FV (+ 0.33, *p* < 0.01). One-year net intervention effects were not observed for self-reported or objective measure of caregivers’ FV intake. One net effect on the household—availability of FV in the home (+ 0.15, *p* < 0.01)—was maintained, but the positive net effect on household food security did not continue into the spring (OR 1.27, *p* = 0.133). At one-year follow-up, 29 caregivers (9.5%) reported purchasing a winter CSA; both sensitivity analyses to explore this source of potential bias produced results consistent with the tabled data.

**Table 3:** Comparison of one-season and one-year net effects of the F3HK intervention on self-reported outcomes, 2016-2018

	One-season Change Net Intervention Effect (*n*=305)		One-year Change Net Intervention Effect (*n*=305)	
	Adjusted Estimate	Sig./CI	Adjusted Estimate	Sig./CI
**PRIMARY OUTCOMES**				
**Child’s FV Intake** (cups/day)				
Total, NCI-FVS	+0.56	0.20	+0.20	0.60
Total without Juice, NCI FVS	+0.58	0.09	+0.20	0.51
**Child’s Intake of SSBs and Processed Snacks** (times/month)				
Sweets, BSQ2	+7.50	0.19	+3.52	0.53
Salty Snacks, BSQ2	-0.37	0.93	+1.20	0.76
SSBs, BSQ2	-0.07	0.99	-2.14	0.73
**SECONDARY OUTCOMES**				
**Child’s Physical Activity** (days/week)	+0.09	0.69	-0.02	0.95
**Child’s Sedentary Behavior** (hours/day)				
Time Watching TV on a School Day	-0.17	0.19	-0.09	0.40
Time Playing Video Games on a School Day	+0.01	0.89	-0.06	0.46
**Caregiver’s Ability to Select, Store, and Prepare CSA Produce**				
Cooking Techniques and Meal Preparation Self-Efficacy, original 14-item (scale 1-5)	+0.20	**<0.01**	+0.28	**<0.01**
Cooking Techniques and Meal Preparation Self-Efficacy, expanded 21-item (scale 1-5)	+0.29	**<0.01**	+0.34	**<0.01**
**Caregiver’s Ability to Substitute FV for Energy-Dense Foods** (times/month)				
Preparing FV as Snacks for Children	+19.22	**<0.01**	+9.16	0.11
Preparing Fruit as Snacks for Children	+7.89	**<0.01**	+2.48	0.28
Preparing Vegetables as Snacks for Children	+12.09	**0.04**	+6.88	0.14
**Caregiver’s Knowledge, Attitudes, and Beliefs About FV**				
Knew Adult FV Recommendation Was 5+ Cups/Day (%, OR)	1.16	0.89, 1.51	1.13	0.86, 1.48
Knew FV Recommendation Was ≥ Half of Dinner Plate (%, OR)	1.29	0.90, 1.86	1.29	0.91, 1.83
General Nutrition Knowledge Belief (scale 1-4)	-0.08	0.11	-0.08	0.065
**Negative** Cooking Attitudes (scale 1-5)	-0.35	**<0.01**	-0.25	**<0.01**
Self-Efficacy for Eating and Cooking Fruits and Vegetables (scale 1-5)	+0.38	**<0.01**	+0.33	**<0.01**
**Caregiver’s FV Intake** (cups/day)				
Total, NCI-FVS	+1.10	**0.02**	+0.72	0.10
Total without Juice, NCI FVS	+1.01	**0.03**	+0.64	0.08
**Caregiver’s Intake of SSBs and Processed Snacks** (times/month)				
Sweets, BSQ2	-6.78	0.30	-2.95	0.56
Salty Snacks, BSQ2	-2.31	0.52	-1.70	0.58
SSBs, BSQ2	-9.61	0.22	-10.01	0.11
**Availability and Accessibility of FV in the Home**				
Availability of FV in the Home (scale 1-4)	+0.11	**0.02**	+0.15	**<0.01**
Accessibility of FV in the Home (scale 1-4)	+0.11	0.20	+0.10	0.14
How Easy to Afford FV (Likert-type 1-5)	+0.12	0.27	+0.07	0.45
How Easy to Access FV (Likert-type 1-5)	+0.23	**0.03**	+0.16	0.10
**Household is Food Secure, FFSM** (OR)	1.67	**1.18, 2.37**	1.27	0.93, 1.72†

Significance of intervention status on change in outcome from baseline was tested using multiple linear regression for continuous variables and multiple logistic regression for dichotomous variables, adjusted for baseline value of the dependent variable

Durability was assessed by including an interaction term for intervention and season in a mixed model that also included year of entry and baseline value of the outcome and ID number as a random effect

† Significant interaction term indicates difference in net intervention effect at T2 relative to T1 (*p* <0.05)

Within the intervention group, the number of weeks that the CSA share was picked-up was associated with three positive one-season changes: one additional week of CSA share pick-up was associated with a decrease in child’s salty snack intake (− 0.60, *p* < 0.05) and an increase in adult’s FV intake without juice (+ 0.12, *p* < 0.01; Table 4). In addition, one additional week of CSA pick up was associated with improved household food security (+ 0.09 Ln odds, *p* < 0.05); for example, households that picked up their share 21 weeks (75th percentile) were 3.73 times as likely to be food secure as households that picked up only 7 weeks (25th percentile). An increase in number of lessons attended was associated with short-term reduction in accessibility of FV in the home (− 0.03, *p* < 0.05).

**Table 4:** Associations between one-season change in outcomes and participation dose among F3HK intervention group members, 2016-2017

	Weeks of CSA Share Pick-up			Number of Lessons Attended		
	n	Adjusted Estimate	Sig.	n	Adjusted Estimate	Sig.
**PRIMARY OUTCOMES**						
**Child’s FV Intake** (cups/day)						
Total, NCI-FVS	99	+0.02	0.71	116	+0.07	0.40
Total without Juice, NCI-FVS	99	+0.01	0.86	116	+0.00	0.94
Total, ASA24	89	+0.00	0.85	108	+0.02	0.53
Total without Juice, ASA24	89	-0.01	0.70	108	+0.03	0.44
**Child’s Mean Skin Carotenoid RRS Score**	79	+41.04	0.80	100	-150.26	0.59
**Child’s Intake of SSBs and Processed Snacks**						
Sweets (times/month), BSQ2	100	+0.11	0.80	119	+1.39	0.08
Salty Snacks (times/month), BSQ2	100	-0.60	**0.02**	119	-0.74	0.15
SSBs (times/month), BSQ2	100	+0.63	0.39	119	+1.28	0.33
Solid Fat Intake (g), ASA24	89	-0.13	0.59	108	+0.30	0.48
Sodium Intake (mg), ASA24	89	-1.80	0.90	108	+33.93	0.15
Added Sugar Intake (tsp), ASA24	89	-0.11	0.27	108	+0.02	0.91
**Child’s Overall Diet Quality**						
Total HEI Score, ASA24	89	-0.65	0.15	108	+0.06	0.94
Energy as %EER^a^, ASA24	89	+0.15	0.39	108	+0.14	0.66
**SECONDARY OUTCOMES**						
**Child’s BMI-for-age** (percentile)	98	-0.25	0.25	119	+0.63	0.10
**Child’s Physical Activity** (days/week)	100	-0.01	0.69	118	-0.07	0.20
**Child’s Sedentary Behavior** (hours/day)						
Time Watching TV on a School Day	100	+0.00	0.84	119	+0.03	0.12
Time Playing Video Games on a School Day	100	-0.02	0.15	119	+0.00	0.99
**Caregiver’s Ability to Select, Store, and Prepare CSA Produce**						
Cooking Techniques and Meal Preparation Self-Efficacy, original 14-item (scale 1-5)	100	+0.01	0.27	119	+0.02	0.11
Cooking Techniques and Meal Preparation Self-Efficacy, expanded 21-item (scale 1-5)	100	+0.01	0.24	119	+0.02	0.08
**Caregiver’s Ability to Substitute FV for Energy-Dense Foods** (times/month)						
Preparing FV as Snacks for Children	100	-0.05	0.94	119	-1.03	0.33
Preparing Fruit as Snacks for Children	100	+0.07	0.80	119	-0.53	0.30
Preparing Vegetables as Snacks for Children	100	-0.07	0.86	119	-0.53	0.49
**Caregiver’s Knowledge, Attitudes, and Beliefs About FV**						
Knew Adult FV Recommendation Was 5+ Cups/Day	100	+0.06	0.10	119	+0.09	0.15†
Knew FV Recommendation Was ≥ Half of Dinner Plate	100	+0.07	0.12	117	+0.12	0.24
General Nutrition Knowledge Belief (scale 1-4)	99	+0.00	0.35	118	+0.01	0.10
**Negative** Cooking Attitudes (scale 1-5)	100	+0.00	0.71	119	-0.01	0.51
Self-Efficacy for Eating and Cooking Fruits and Vegetables (scale 1-5)	100	+0.00	0.73	119	+0.01	0.78
**Caregiver’s FV Intake** (cups/day)						
Total, NCI-FVS	98	+0.08	0.10	116	+0.02	0.80
Total without Juice, NCI-FVS	98	+0.12	**<0.01**	116	+0.06	0.49
**Caregiver’s Mean Skin Carotenoid RRS Score**	81	+287.68	0.10	102	-71.16	0.81
**Caregiver’s Intake of SSBs and Processed Snacks** (times/month)						
Sweets, BSQ2	100	-0.96	0.15	118	-1.42	0.23
Salty Snacks, BSQ2	100	-0.02	0.94	118	-0.33	0.40
SSBs, BSQ2	100	-0.04	0.96	118	-0.22	0.85
**Availability and Accessibility of FV in the Home**						
Availability of FV in the Home (scale 1-4)	99	+0.00	0.56	118	-0.00	0.87
Accessibility of FV in the Home (scale 1-4)	98	-0.01	0.41	117	-0.03	**0.04**
How Easy to Afford FV (Likert-type 1-5)	100	+0.02	0.07	119	-0.00	0.98
How Easy to Access FV (Likert-type 1-5)	100	+0.01	0.25	119	+0.02	0.35
**Household is Food Secure, FFSM** (ln odds)	99	+0.09	**0.04**	117	-0.04	0.63

Significance of dose effect on change in outcome from baseline was tested using multiple linear regression for continuous variables and multiple logistic regression for dichotomous variables, adjusted for baseline value of the dependent variable

Aforementioned, participants who received monthly SNAP benefits were able to use them to pay for their portion of the CSA share price, and 24% of participants used SNAP benefits all or most weeks. Of the 62% who never used SNAP benefits to make a weekly CSA payment, 77% indicated this was because they did not receive SNAP benefits.

## Discussion

The F3HK trial addressed gaps in our understanding of how CO-CSA affects dietary intake and related practices in low-income households with children, and strengthened the methodology used in examining CSA impacts. Overall, we found that CO-CSA plus nutrition education modestly improved caregivers’ FV intake across one season of participation and had no effect on children’s FV intake. Almost one-half (47%) of enrolled children and almost one-third (30%) of caregivers met FV intake recommendations at baseline; those levels are higher than the U.S. population overall in which less than 10% of children and adults meet vegetable recommendations, and somewhat more meet fruit recommendations (40% of children and 12% of adults) [2, 84]. Enrolled caregivers also were more likely to have a college education or graduate degree than the low-income population overall in the target counties (48.9% vs 17.8%) [85]. Given education and the fact that enrolled caregivers had to agree to use their own cash or SNAP benefits to purchase the CO-CSA every week throughout the growing season, the program may have attracted families that were atypical in their orientation toward health. There is other evidence that suggests CO-CSA programs attract a subset of the population, low-income or otherwise, for whom FV access and intake is atypically positive. For example, a descriptive study with low-income families found that CO-CSA members reported significantly higher FV intake for themselves and their children than US averages, and they and their children more often met recommendations for vegetable intake than the overall US population [31]. Two studies reported that households participating in CO-CSA had greater access to or availability of FV at baseline [29, 37]: one of these targeted low-income participants [37] and the other did not [29]. A third study reported that CO-CSA applicants had greater access to and availability of FV relative to a no-CSA comparison sample regardless of whether the applicants actually participated [30]. Although a greater percent of participants in this study met FV guidelines than the general public, the majority of study participants and the general US adult population are not meeting FV intake guidelines [2]. People with lower incomes are even less likely to meet FV recommendations [2]. Future research is needed to determine ways to attract any individuals who are at-risk of low FV intake to CSA, and to make CSA more inclusive and acceptable to households with the greatest financial need.

The sample’s atypical baseline FV intake may help to explain why the intervention had no effect on children’s FV intake and only a modest effect on caregivers’ FV intake, although two-thirds of children and three-quarters of caregivers did not meet FV recommendations at baseline, suggesting that increases in FV intake were still needed to support health. An additional explanation may be that intervention-targeted behaviors may shift first among caregivers, who were more active in intervention activities, and that their behavioral modeling of FV intake and other related outcomes may take longer to manifest in measurable behavior change among their children [86]. This is an important avenue for future research.

F3HK was successful in improving caregivers’ cooking attitudes, self-efficacy related to preparing meals and FV specifically, and the frequency with which caregivers prepared FV as snacks for their children. Notably, most of these improvements were maintained into the following spring, well after the summer CSA season had ended. These findings are in contrast with a pilot study (*n* = 9) that found no change in self-efficacy to eat FV or attitudes toward food preparation after CSA participation [29]. The success of the current intervention may be due, in part, to the incorporation of educational materials (available in group classes or online). However, lesson attendance was not found to be associated with positive change in outcomes among the intervention group. Participants were invited, but not required, to attend the nine in-person, CSA-tailored nutrition education classes. Although 77% of caregivers and 54% of children attended at least one class [43], overall attendance rates were low. Conversely, the number of weeks that the CSA share was picked-up was associated with a few positive, one-season changes within the intervention group (decrease in child’s salty snack intake and increase in adult’s FV intake without juice).

F3HK was also successful in improving household food security status, which is notable given that more than half of the families in this study reported food insecurity or participation in a federal food benefit program (WIC and/or SNAP) in the month prior to study enrollment. However, this change was not maintained into the following spring; that is, after the CSA ended, household food security reverted to levels observed before the intervention. This suggests that the modest monetary subsidy for the CSA had a meaningful effect on food security during the active intervention period. Two other studies examined CO-CSA participation in association with food security status; both reported little to no association [22, 27].

The present study successfully addressed some of the limitations of prior CO-CSA studies which had small samples [27, 29, 31, 37], no comparison group [27, 29, 31, 34], included only data from during/after the CSA season [30, 31, 33], and/or recruited samples that did not prioritize low-income participants [33, 34]. F3HK had a sufficiently-powered sample, employed a randomized-controlled trial design with longitudinal data collection, and prioritized recruitment of low-income families in four geographically distinct parts of the U.S. In addition, we uniquely report on CO-CSA effects on the diet quality of more than one household member. Finally, we included an objective measure of FV intake to support self-reported data.

Despite these strengths, three limitations of this study deserve note. First, low participation in the CSA-tailored education diluted intervention fidelity and may have hindered effectiveness. Second, self-selection into this lengthy intervention trial that required on-going expenditure of cash or benefits resulted in selection bias. As previously mentioned, enrolled caregivers were more educated, and both adults and children had higher than expected FV intake at baseline. Participants were representative of the racial distribution of the communities in which they lived [87] yet were predominantly white. As such, households enrolled in this trial share characteristics with the middle- and upper-income households included in most other CSA studies [9–14, 16, 17, 20, 21, 23–25, 33], and may not be generalizable to the population at large. However, the findings are useful for understanding the impact of this intervention on families motivated to commit to a CSA but without the financial resources needed to participate. Third, the net effects of the intervention were estimated largely from self-reported outcome data. Self-reported data, and particularly dietary intake, are prone to recall bias [88, 89] and social desirability bias [90] which may have skewed results. However, positive effects on caregivers’ FV intake were corroborated by corresponding effects on their objectively-assessed skin carotenoids thereby reducing concerns about reporting bias.

Rigorously examining participation in CO-CSA plus nutrition education and its impact in real-world settings is timely and necessary given interest in interventions like CO-CSA to address access to healthy foods as a social determinant of health. In a recent survey of Medicaid Medical Director Network members, the most commonly reported screening topics were housing instability and food insecurity [91], suggesting that cost-effective and impactful interventions will be needed to address such social determinants of health. Future studies of CO-CSAs should employ pragmatic trials to compare the effectiveness of CO-CSA to other promising interventions for improving FV intake, reducing food insecurity, and addressing health disparities.

## Conclusion

Our study suggests that subsidized CSA memberships plus nutrition education are a viable approach to improve caregiver FV intake, caregiver behaviors and confidence around serving FV, and household food security for low-income families, although seasonality of most CSAs may limit their potential to improve year-round food security.

## Supplementary Information

**Additional file 1: Supplemental Table 1.** One-season net effects of F3HK among enrollees with complete data in four U.S. states, 2016–2017.

## Data Availability

The datasets used and analyzed during the current study are available from the corresponding author on reasonable request.

## Abbreviations

BMI: Body Mass Index
BSQ2: Beverage and Snack Questionnaire 2
CO-CSA: Cost-offset Community Supported Agriculture
CSA: Community Supported Agriculture
EER: Estimated Energy Requirements
F3HK: Farm Fresh Foods for Healthy Kids
FCS: Fully conditional specification
FV: Fruit and vegetables
FVS: Fruit and Vegetable Screener
HEI: Healthy Eating Index
ITT: Intent-to-treat
KAB: Knowledge, attitudes, and behaviors
MAR: Missing at random
NCI: National Cancer Institute
RRS: Resonance Raman spectroscopy
SSB: Sugar sweetened beverage
