# Supplementary material for: Community supported agriculture plus nutrition education improves skills, self-efficacy, and eating behaviors among low-income caregivers but not their children: a randomized controlled trial
Source: Int J Behav Nutr Phys Act. 2021 Aug 31;18:112. doi: 10.1186/s12966-021-01168-x (PMC8406558; doi:10.1186/s12966-021-01168-x)
Supplement: Supplementary file 1 — Additional file 1: Supplemental Table 1. One-season net effects of F3HK among enrollees with complete data in four U.S. states, 2016–2017. [file 12966_2021_1168_MOESM1_ESM.docx]

Supplemental Table 1. One-season effects among enrollees with complete data in four U.S. states, 2016-2017

|  | **Intervention** | | | **Control** | | | **Net Effect After Adjustment for Baseline** | **Sig./CI** |
| --- | --- | --- | --- | --- | --- | --- | --- | --- |
|  |  | **Baseline** | **One-season Change** |  | **Baseline** | **One-season Change** |  |  |
|  | ***n*** | **Mean/%** | **Mean/%** | ***n*** | **Mean/%** | **Mean/%** |  |  |
| **PRIMARY OUTCOMES** |  |  |  |  |  |  |  |  |
| **Child’s FV Intake** (cups/day) |  |  |  |  |  |  |  |  |
| Total, NCI-FVS | 116 | 4.13 | -0.19 | 105 | 3.34 | -0.24 | +0.50 | 0.14 |
| Total without Juice, NCI-FVS | 116 | 3.08 | -0.02 | 105 | 2.64 | -0.26 | +0.48 | 0.07 |
| Total, ASA24 | 108 | 2.95 | 0.16 | 97 | 2.83 | -0.04 | +0.30 | 0.10 |
| Total without Juice, ASA24 | 108 | 2.57 | 0.20 | 97 | 2.47 | -0.07 | +0.35 | **0.05** |
| **Child’s Mean Skin Carotenoid RRS Score** | 100 | 37588.25 | -1652.66 | 91 | 36468.15 | -1616.17 | +187.93 | 0.87 |
| **Child’s Intake of SSBs and Processed Snacks** |  |  |  |  |  |  |  |  |
| Sweets (times/month), BSQ2 | 119 | 34.33 | -4.21 | 106 | 39.00 | -12.16 | +5.93 | 0.23 |
| Salty Snacks (times/month), BSQ2 | 119 | 23.27 | 0.71 | 106 | 25.09 | -0.12 | -0.97 | 0.78 |
| SSBs (times/month), BSQ2 | 119 | 39.25 | -3.08 | 106 | 39.47 | -6.31 | +3.24 | 0.58 |
| Solid Fat Intake (g), ASA24 | 108 | 32.72 | 0.71 | 97 | 30.58 | 2.25 | +-0.46 | 0.82 |
| Sodium Intake (mg), ASA24 | 108 | 2702.40 | 144.13 | 97 | 2659.84 | 41.07 | +102.11 | 0.37 |
| Added Sugar Intake (tsp), ASA24 | 108 | 12.04 | -0.78 | 97 | 10.83 | 0.54 | -1.21 | 0.11 |
| **Child’s Overall Dietary Quality** |  |  |  |  |  |  |  |  |
| Total HEI Score, ASA24 | 108 | 115.70 | -1.41 | 97 | 115.99 | -0.71 | -0.84 | 0.82 |
| Energy as %EER^a^, ASA24 | 108 | 60.55 | -0.54 | 97 | 60.83 | -1.40 | +0.72 | 0.60 |
| **SECONDARY OUTCOMES** |  |  |  |  |  |  |  |  |
| **Child’s BMI-for-age** (percentile) | 119 | 65.80 | -0.57 | 106 | 71.05 | 0.22 | -1.10 | 0.51 |
| **Child’s Physical Activity** (days/week) | 118 | 5.52 | 0.28 | 106 | 5.08 | 0.58 | -0.04 | 0.84 |
| **Child’s Sedentary Behavior** (hours/day) |  |  |  |  |  |  |  |  |
| Time Watching TV on a School Day | 119 | 1.28 | -0.24 | 106 | 1.28 | -0.09 | -0.15 | 0.16 |
| Time Playing Video Games on a School Day | 119 | 0.75 | -0.15 | 105 | 0.73 | -0.15 | +0.02 | 0.85 |
| **Caregiver’s Ability to Select, Store, and Prepare CSA Produce** |  |  |  |  |  |  |  |  |
| Cooking Techniques and Meal Preparation Self-Efficacy, original 14-item (scale 1-5) | 119 | 3.93 | 0.22 | 107 | 3.77 | 0.05 | +0.23 | **<0.01** |
| Cooking Techniques and Meal Preparation Self-Efficacy, expanded 21-item (scale 1-5) | 119 | 3.75 | 0.31 | 107 | 3.56 | 0.06 | +0.31 | **<0.01** |
| **Caregiver’s Ability to Substitute FV for Energy-Dense Foods** (times/month) |  |  |  |  |  |  |  |  |
| Preparing FV as Snacks for Children | 119 | 68.71 | 16.29 | 107 | 65.49 | -2.14 | +19.78 | **<0.01** |
| Preparing Fruit as Snacks for Children | 119 | 38.85 | 5.63 | 107 | 34.72 | 0.10 | +7.67 | **<0.01** |
| Preparing Vegetables as Snacks for Children | 119 | 29.86 | 10.65 | 107 | 30.77 | -2.24 | +13.19 | **<0.01** |
| **Caregiver’s Knowledge, Attitudes, and Beliefs About FV** |  |  |  |  |  |  |  |  |
| Knew Adult FV Recommendation Was 5+ Cups/Day | 119 | 47.9 | 5.0 | 107 | 34.6 | 6.5 | 1.18 | 0.89, 1.56 |
| Knew FV Recommendation Was ≥ Half of Dinner Plate | 117 | 76.1 | 10.2 | 105 | 74.3 | 6.7 | 1.22 | 0.84, 1.79 |
| General Nutrition Knowledge Belief (scale 1-4) | 118 | 3.23 | -0.02 | 107 | 3.16 | 0.07 | -0.07 | 0.12 |
| **Negative** Cooking Attitudes (scale 1-5) | 119 | 2.09 | -0.22 | 107 | 2.23 | 0.11 | -0.36 | **<0.01** |
| Self-Efficacy for Eating and Cooking Fruits and Vegetables (scale 1-5) | 119 | 3.75 | 0.35 | 107 | 3.65 | -0.04 | +0.44 | **<0.01** |
| **Caregiver’s FV Intake** (cups/day) |  |  |  |  |  |  |  |  |
| Total, NCI-FVS | 116 | 4.30 | 0.09 | 104 | 3.63 | -0.22 | +0.81 | **0.04** |
| Total without Juice, NCI-FVS | 116 | 3.74 | 0.11 | 104 | 3.23 | -0.36 | +0.91 | **0.01** |
| **Caregiver’s Mean Skin Carotenoid RRS Score** | 102 | 32153.74 | 1266.64 | 94 | 31313.26 | -1732.26 | +3129.05 | **<0.01** |
| **Caregiver’s Intake of SSBs and Processed Snacks** (times/month) |  |  |  |  |  |  |  |  |
| Sweets, BSQ2 | 118 | 34.00 | -10.21 | 107 | 28.39 | -3.38 | -5.98 | 0.17 |
| Salty Snacks, BSQ2 | 118 | 14.34 | -0.75 | 107 | 18.88 | -2.73 | -1.32 | 0.57 |
| SSBs, BSQ2 | 118 | 38.39 | -3.32 | 107 | 41.73 | -0.91 | -5.54 | 0.30 |
| **Availability and Accessibility of FV in the Home** |  |  |  |  |  |  |  |  |
| Availability of FV in the Home (scale 1-4) | 118 | 3.48 | 0.12 | 107 | 3.39 | 0.06 | +0.10 | **0.02** |
| Accessibility of FV in the Home (scale 1-4) | 117 | 3.40 | 0.17 | 107 | 3.21 | 0.11 | +0.14 | **0.03** |
| How Easy to Afford FV (Likert-type 1-5) | 119 | 2.82 | 0.26 | 107 | 2.66 | 0.22 | +0.10 | 0.27 |
| How Easy to Access FV (Likert-type 1-5) | 119 | 3.94 | 0.32 | 107 | 3.85 | 0.16 | +0.21 | **0.03** |
| **Household is Food Secure, FFSM** (%, OR) | 117 | 43.6 | 12.0 | 107 | 43.9 | -4.6 | 1.72 | **1.21, 2.45** |
| Significance of intervention status on change in outcome from baseline was tested using multiple linear regression for continuous variables and multiple logistic regression for dichotomous variables. All p-values presented are from models adjusted for the baseline value of the dependent variable.  ^a^ Calculated using baseline age and assuming a moderate level of physical activity | | | | | | | | |
